# The Evolution of Deep Brain Stimulation in Psychiatry: Historical Targets and Network Expansion

**DOI:** 10.1192/j.eurpsy.2026.11748

**Published:** 2026-07-31

**Authors:** O. Scheuermann, D. Valle, E. Jetter, C. Angelle, B. Carr

**Affiliations:** 1 Department of Psychiatry; 2 College of Medicine, University of Florida, Gainesville, United States

## Abstract

**Introduction:**

Deep Brain Stimulation (DBS) has transformed the management of treatment-resistant psychiatric disorders, originating as a therapeutic approach for movement disorders before expanding into psychiatry. Initially developed to alleviate motor symptoms in Parkinson’s disease, DBS has since been adapted for psychiatric conditions, particularly obsessive-compulsive disorder (OCD), major depressive disorder (MDD), and Tourette’s syndrome. This poster traces the historical evolution of DBS in psychiatry, emphasizing the transition from focal targeting to network-centric modulation.

**Objectives:**

To trace the historical evolution of psychiatric DBS, highlighting the transition from early focal targets to network-centric approaches and identifying future directions in precision neuromodulation.

**Methods:**

A review of historical milestones, target development, and network models was conducted. Key stages in the evolution of DBS were examined, including early lesion-based approaches, adoption of new anatomical targets, and the integration of functional neuroimaging and electrophysiological insights into circuit-level modulation.

**Results:**

Initial psychiatric DBS applications targeted the anterior limb of the internal capsule (ALIC) based on lesion studies in OCD. Refinements included the ventral capsule/ventral striatum (VC/VS) to optimize reward and motivational circuitry, the nucleus accumbens (NAc) for compulsive and anhedonic states, and the subthalamic nucleus (STN) adapted from movement disorder treatments. Advances in functional imaging revealed the importance of the cortico-striato-thalamo-cortical (CSTC) loop and broader network dysfunction, shifting DBS strategies toward modulation of large-scale circuits. Milestones include: (1) introduction of DBS for OCD in 1999; (2) adoption of VC/VS targeting in 2004; (3) expansion to STN for depression and OCD in 2008; (4) growing prominence of network-centric models after 2010; and (5) development of closed-loop DBS systems guided by spectral biomarkers since 2022. Emerging targets include the amygdala for PTSD, nucleus accumbens for substance use disorders, and hypothalamus/insula for anorexia nervosa.

**Conclusions:**

The evolution of DBS in psychiatry illustrates a shift from static, focal intervention to dynamic, network-level modulation. Understanding this historical trajectory provides essential context for future developments in precision neuromodulation, where therapeutic efficacy will depend not only on target selection but on real-time engagement with the brain’s complex network architecture.

**Disclosure of Interest:**

None Declared

